# Chronological Patterns of Comorbidities in Alzheimer's Disease and Vascular Dementia: A Longitudinal Network Analysis

**DOI:** 10.1002/alz70860_103134

**Published:** 2025-12-23

**Authors:** Chloe Walsh, Antigone Fogel, Vanessa Mabiala, Ramin Nilforooshan, Payam Barnaghi

**Affiliations:** ^1^ Imperial College London, London, United Kingdom; ^2^ UK Dementia Research Institute, Care Research and Technology Centre, Imperial College London, London, United Kingdom; ^3^ Surrey and Borders Partnership NHS Foundation Trust, Leatherhead, United Kingdom; ^4^ UK Dementia Research Institute, Care Research and Technology Centre, London, United Kingdom

## Abstract

**Background:**

Pre‐existing, long‐term health conditions are highly prevalent in people living with dementia, influencing disease progression and care outcomes. However, the timing of when these conditions first occur with respect to onset of Alzheimer's Disease and Vascular Dementia remains unclear. Given that individuals' health and quality of life, are impacted by these pre‐existing conditions decades before a diagnosis of dementia, it is likely they play an important role facilitating onset and progression of the disease. This study sets out to map conditions over time to distinguish patterns unique to Alzheimer's Disease and Vascular Dementia compared to matched controls.

**Method:**

We analysed UK Biobank data, of hospital inpatient ICD‐10 codes from 10,696 participants, with records spanning a median of 16.87 years (IQR = 10.01 years) for participants with dementia and 13.85 years (IQR = 12.65) for controls. A Mann‐Whitney U test was employed to identify significantly different (*p* < 0.05) conditions between dementia and control cohorts. Network analysis was conducted across time frames from 20 years before to 10 years after dementia diagnosis, to visualise the chronological emergence of conditions. Conditions that were statistically significant and exclusive to dementia networks were identified for Alzheimer's Disease and Vascular Dementia, independently. Logistic regression, adjusted for age at diagnosis of dementia and sex, estimated odds ratios for each condition.

**Result:**

Preliminary results suggest that Vascular Dementia exhibits a broader and more distinct comorbidity profile compared to Alzheimer's Disease. Endocrine disorders, particularly type 1 diabetes, were uniquely associated with both dementia sub‐types, in the years preceding diagnosis but, were more prominent in Vascular Dementia. Circulatory diseases, including cerebral infarctions were consistently associated with Vascular Dementia, up to 20 years before diagnosis. In contrast, Alzheimer's Disease showed a less distinct pattern, though mental health conditions emerged as a notable early feature.

**Conclusion:**

These initial findings suggest that Vascular Dementia presents a stronger and more specific comorbidity trajectory than Alzheimer's Disease. Certain conditions may serve as early indicators of specific dementia subtypes, presenting opportunities for targeted screening and preventative measures. Further research is needed to assess the predictive value of these conditions for screening or risk identification.

